# Evaluating postmortem tongue fluids as a tool for monitoring PRRSV and IAV in the post-wean phases of swine production

**DOI:** 10.1186/s40813-025-00432-x

**Published:** 2025-04-07

**Authors:** Onyekachukwu Henry Osemeke, Isadora Machado, Mafalda Mil-Homens, Grant Allison, Michael Paustian, Daniel C. L. Linhares, Gustavo S. Silva

**Affiliations:** 1 Veterinary Diagnostic and Production Animal Medicine, College of Veterinary Medicine, Iowa State University, 2231 Lloyd, 1809 S Riverside Dr., Ames, IA 50011-3619 USA; 2 Walcott Veterinary Clinic, Walcott, IA USA; 3 Paustian Enterprises, Walcott, IA USA

**Keywords:** Swine, Tongue, Tissue fluids, PRRSV, Influenza, RT-qPCR, Wean to finish, Pool

## Abstract

**Background:**

Porcine reproductive and respiratory syndrome virus (PRRSV) and influenza A virus (IAV) are swine pathogens that can significantly impact the performance of post-weaning pigs. While oral fluid (OF) samples are widely used for monitoring these viruses, postmortem tongue fluid (TF) samples present a cost-effective alternative with potential advantages in viral detection. This study aimed to compare the performance of TF and OF samples collected from nursery and finishing pig herds in detecting PRRSV and IAV using reverse transcription-quantitative polymerase chain reaction (RT-qPCR). A Bayesian latent class model was used to estimate diagnostic sensitivity and specificity for TF and OF under the assumption of conditional independence. The study also examined the relationship between mortality rates and RT-qPCR outcomes, the success rate of Sanger sequencing for the PRRSV ORF-5 region, and the effect of pooling daily aggregated TF samples on the probability of PRRSV detection.

**Results:**

IAV was detected in 34.9% of OF samples and 30.2% of TF samples, while PRRSV was identified in 67.4% of OF and 53.5% of TF samples. TF samples had a significantly lower mean Ct for PRRSV (29.1) compared to OF samples (32.8) but had a similar Ct (30.9) to OF (29.7) for IAV. The hierarchical latent class Bayesian model estimated the sensitivity and specificity values for OF as 37.3% and 61.7% for IAV, and 64.3% and 35.1% for PRRSV. The estimated sensitivity and specificity values for TF were 33.5% and 66.0% for IAV, and 53.0% and 47.0% for PRRSV. Among 22 matched TF and OF pairs submitted for PRRSV sequencing, 45.5% of OF samples and 63.6% of TF samples were successfully sequenced, with the higher success rate for TF attributed to having lower Ct values. Additionally, mortality rates were notably higher when PRRSV was detected, especially in cases with concurrent IAV detection. Regarding sample pooling, our results indicated that pooling TF samples significantly increased detection probabilities, with a 1/7 dilution achieving a 79% RT-qPCR detection rate, compared to a detection rate of 14.3% when testing a single day's TF sample from a week with only one positive day.

**Conclusion:**

The findings support the use of TF samples as a viable complement or alternative to OF samples for PRRSV and IAV surveillance in post-weaning pigs when mortalities are available. The cost-efficiency of TF sampling can enhance monitoring compliance, improve early pathogen detection, and facilitate timely responses to emerging threats in swine production. This study advocates for the adoption of TF as a risk-based sampling strategy in nursery and grow-finish settings, complementing live animal samples such as OF, ultimately contributing to better herd health management.

**Supplementary Information:**

The online version contains supplementary material available at 10.1186/s40813-025-00432-x.

## Background

Porcine reproductive and respiratory syndrome virus (PRRSV) and influenza A virus (IAV) are primary members of the porcine respiratory disease complex [1] and cause fever, inappetence, and respiratory distress in pigs post-weaning [2, 3]. Not many efficient tools are available today for surveilling pigs in the post-weaning phases for the pathogens mentioned above; the oral fluid sample (OF) is the major sample type submitted from post-weaning phases of swine production for PRRSV and IAV surveillance according to the Swine Disease Reporting System, a consortium of six veterinary diagnostic laboratories in the US [4, 5]. The industry-accepted strategy for surveilling growing pig herds for PRRSV RNA is the reverse transcription-quantitative polymerase chain reaction or RT-qPCR of six geospatially distributed OF samples [4]. Even though there is not a similar industry-wide consensus on IAV surveillance strategies in post-weaning phases of swine production, there are a variety of samples (for example, postmortem tissues, swabs, and oral fluids) submitted to US veterinary diagnostic laboratories for this purpose.

The postmortem tongue fluid (TF) sample from nursery mortalities was recently demonstrated to be a cost-efficient, risk-based sample for PRRSV-1 surveillance [5]. TF use has been similarly demonstrated in breeding herds for PRRSV-1 and PRRSV-2 surveillance [5–7]. There is sparse information today on using TF as a risk-based sample for swine pathogen surveillance in nursery and grow-finish sites or on how TF compares with OF for the same purposes. A preliminary proof-of-concept study [8] that evaluated blended tongue tissues, tongue fluids, and variable numbers of OF for PRRSV and IAV surveillance by RT-qPCR in growing herds demonstrated that PRRSV RNA can be found in both sample types, but the referenced study was not designed to compare detection rates between both sample types over time statistically. This study obtained TF by best sampling practice and OF obtained by the industry-accepted strategy for PRRSV surveillance. In addition, the same herds are monitored over time from placement, providing data on the effect of the stage of production on the probability of PRRSV and IAV detection in both sample types. This study also investigated associations between mortality rates and RT-qPCR outcomes (detection rates and Cts) of TF on the mentioned pathogens. Preliminary studies from our team on PRRSV surveillance in breeding herds using TF samples showed that when positive weekly pools of daily collected TF are open for testing, not every sample had a positive RT-qPCR test. Thus, there is also a need to evaluate pooling daily collected TF on the probability of PRRSV detection by RT-qPCR under field conditions. The probability of successful Sanger sequencing of the PRRSV ORF-5 region in TF and OF obtained from growing sites has not been hitherto compared in published literature. The results obtained from the Sanger sequencing comparisons will contribute significantly to addressing knowledge gaps related to the viability of using TF to identify the PRRSV variant present in growing sites, particularly in comparison to the more conventional OF samples.

Evaluating the herd sensitivity of TF compared to OF for disease pathogen surveillance will help swine practitioners understand if-, when-, and how to use this alternative tool for surveilling pigs post-weaning. Mortalities are disposed of daily, and collecting tongue tissues will not significantly increase labor time; in addition, this sample is a risk-based sample with multiple animals represented within a single sample/test. The cost- and labor efficiency proffered by this sample type will improve monitoring compliance in the face of a foreign/emerging animal disease, enhancing early detection and timely response.

Surveillance programs in growing pig herds are generally not as robust as those in the breeding herds, consequently, even a cost-effective sample option does not guarantee best-practice compliance if the investment in surveillance is inadequate. A question addressed in this study was the consequence of pooling daily aggregated tongues by week. The dilution of PRRSV RNA targets in a sample is expected when this sample is pooled with negative samples [9–12] or when media, such as PBS, is added. This process is naturally exponential in PCRs as gene targets ideally double with every cycle. However, the composition of biological samples can affect the integrity of nucleic acids [13] and the efficiency of the PCR process [14]; therefore, to understand the extent to which dilution by pooling affects the RT-qPCR detection of PRRSV in a specific sample type, an experiment is required. Currently, no such information is available in the literature for TF samples.

The primary objective of this study was to compare the RT-qPCR detection rates of PRRSV and IAV in TF and OF in the nursery and grow-finish phases of swine production. The secondary objectives of the study are: (1) to assess the effect of sample type on the successful sequencing of the PRRSV ORF-5 gene; (2) to characterize the implications of pooling TF by week on the probability of PRRSV RT-qPCR detection. The associations between productivity data (mortality) and RT-qPCR detection of viral RNA in both sample types were also evaluated.

## Results

### RT-qPCR outcomes

**Fig. 1:**
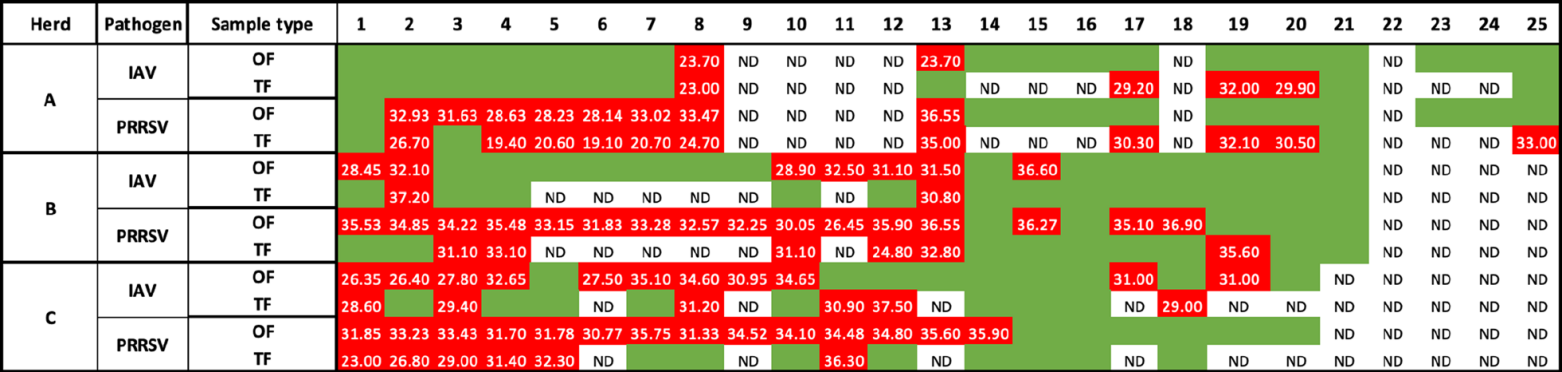
Weekly RT-qPCR detection and mean cycle-threshold values of PRRSV and IAV in oral fluids (OF) and postmortem tongue fluids pools (TF). Green colors indicate negative RT-qPCR tests, red colors indicate at least 1 positive RT-qPCR test with mean Ct values in the cells. ND indicates weeks with no submissions of that sample type

There were 60 weeks with submissions across all three herds. OF was submitted in all 60 weeks, while TF was submitted in only 43 of the 60 weeks (Fig. 1).

**Fig. 2:**
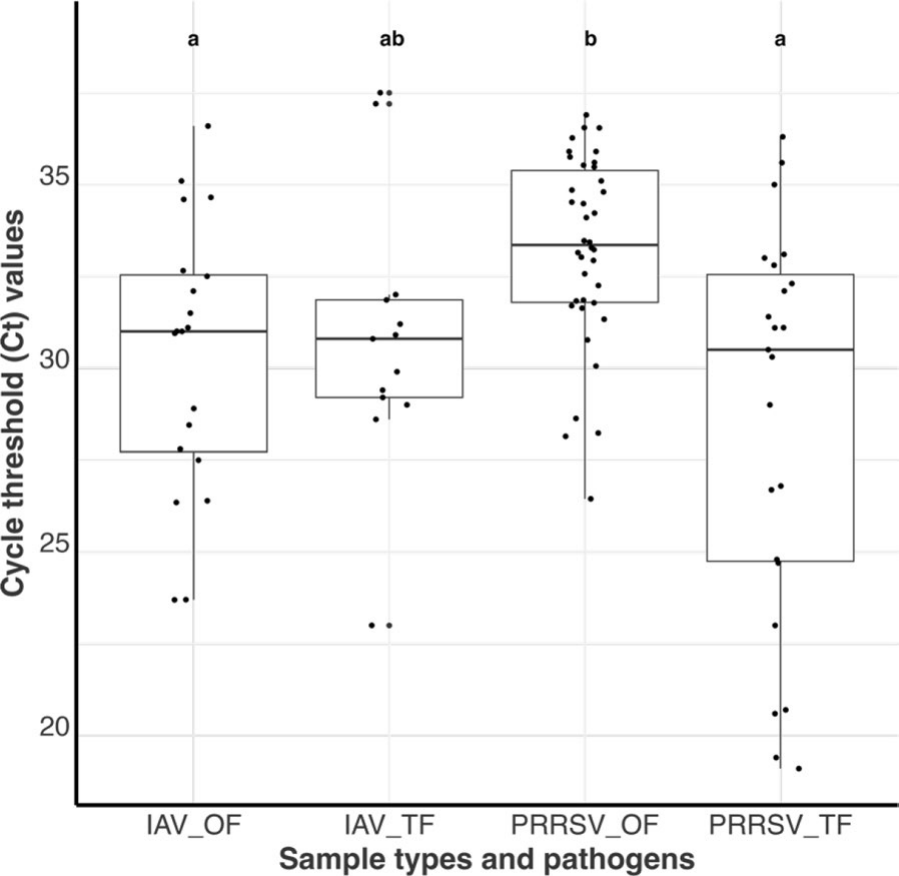
RT-qPCR Cycle threshold value distribution for Influenza A virus in oral fluids (IAV_OF), Influenza A virus in postmortem tongue fluids (IAV_TF), Porcine reproductive and respiratory syndrome virus in oral fluids (PRRSV_OF), and Porcine reproductive and respiratory syndrome virus in postmortem tongue fluids (PRRSV_TF). All samples were submitted from growing pig herds. Distributions with different letters are statistically different (α = 0.05)

Of the 43 weeks with both sample types, IAV was detected in 15 (34.9%) OF and 13 (30.2%) TF samples, while PRRSV was detected in 29 (67.4%) OF and 23 (53.5%) TF samples. PRRSV RT-qPCR least square mean Ct (Fig. 2) in TF samples was statistically lower (*p* = 0.0012) in TF (29.13 [22.37, 35.89]) compared to OF (32.8 [24.74, 40.87]). There was no statistical difference (*p* = 0.771) between the IAV RT-qPCR least square mean Ct in TF samples (30.88 [25.11, 36.64]) compared to OF samples (29.66 [23.26, 36.07]).

### Diagnostic performance assessment

The two-by-two comparisons of PRRSV and IAV detection rates in TF relative to OF are shown in Table 1. The hierarchical latent class Bayesian model estimated moderate diagnostic sensitivity and specificity values for both TF and OF across pathogens. The median (and 95% credible interval) estimates for the modeled parameters are presented in Table 2. All monitored parameters had $\widehat{R}$ values below 1.1 and the trace plots showed good mixing.

**Table 1:** A and B Two-by-two tables comparing the detection (yes or no) by week of PRRSV (A) and IAV (B) in postmortem tongue fluids (TF) samples and oral fluids (OF) samples

A. PRRSV detection				B. IAV detection			
	OF negative	OF positive	Total		OF negative	OF positive	Total
TF negative	9	11	20	TF negative	22	8	30
TF positive	5	18	23	TF positive	6	7	13
Total	14	29	43	Total	28	15	43

**Table 2:** Diagnostic performance (posterior summaries) of oral fluids and postmortem tongue fluids in detecting Influenza A virus and porcine reproductive and respiratory syndrome virus

Pathogen	Sample type	Parameter	Median estimates (95% credible interval)
Influenza A virus (IAV)	Oral fluids (OF)	Sensitivity	37.3% (10.0%, 82.8%)
		Specificity	61.7% (17.3%, 89.6%)
	Postmortem tongue fluids (TF)	Sensitivity	33.5% (8.1%, 82.2%)
		Specificity	66.0% (18.0%, 91.8%)
Porcine reproductive and respiratory syndrome virus (PRRSV)	Oral fluids (OF)	Sensitivity	64.3% (23.7%, 88.0%)
		Specificity	35.1% (11.6%, 75.0%)
	Postmortem tongue fluids (TF)	Sensitivity	53.0% (14.1%, 88.0%)
		Specificity	47.0% (11.8%, 84.7%)

### PRRSV sanger sequencing

Of the 22-week-matched OF and TF samples submitted for PRRSV ORF-5 sequencing, 10 were from herd A, 6 from herd B, and 6 from herd C (Additional file 2: Table S1). For herd A, 7/10 of the OF were successfully sequenced with two variants (1-4-4 L1A, 1-2-4 L1A) identified, while 7/10 of the TF were successfully sequenced with only one PRRSV variant (1-4-4 L1A) identified. For herd B, 2/6 of the OF were successfully sequenced with two variants (1-3-2 L8C, 1-1-2 L8C) identified, while 4/6 of the TF were successfully sequenced with two variants (1-3-2 L8C, 1-1-2 L8C) identified. For herd C, 1/6 of the OF were successfully sequenced with one variant (1-7-2 L1A) identified, while 3/6 of the TF were successfully sequenced with two variants (1-7-2 L1A, 1-3-2 L8C) identified.

**Fig. 3:**
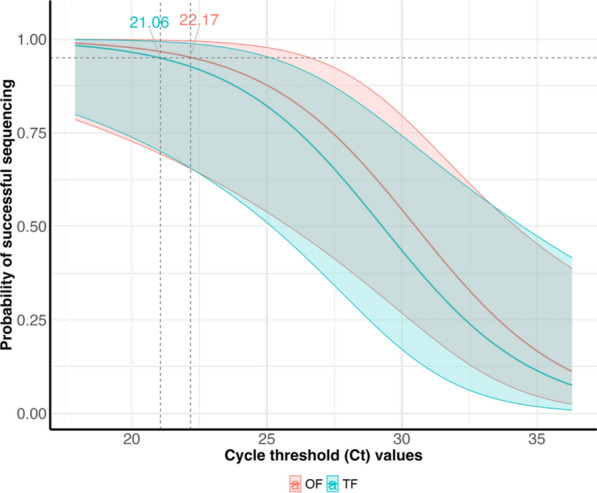
The predicted probability of successful PRRSV ORF-5 Sanger sequencing given the RT-qPCR cycle threshold (Ct) values of tested oral fluids (OF) and postmortem tongue fluids (TF). The horizontal dashed line represents the 95% probability of successful Sanger sequencing. The vertical lines intersecting with the horizontal lines represent the Cts beyond which the 95% probability of successful Sanger sequencing falls below 95% (22.17 for OF, and 21.06 for TF)

Across all three herds, 14 (63.64%) TF samples were successfully sequenced, while only 10 (45.45%) OF samples were successfully sequenced. After adjusting for the Ct values of the samples in the statistical models, there was no statistical difference (*p* = 0.603) in the probability of successfully obtaining the PRRSV ORF-5 sequence in the TF samples (0.54 [0.21, 0.83]) compared to the OF samples (0.64 [0.28, 0.89]). The Ct of the sample, regardless of the sample type, was the significant predictor of the probability of detection. When the predicted probabilities of successful ORF-5 sequencing were plotted against PRRSV RT-qPCR Ct values for both sample types, an OF Ct of 22.17 or lower, or a TF Ct of 21.06 or lower is needed to have a ≥ 95% probability of successful PRRSV ORF-5 sequencing (Fig. 3).

### Pooling

**Fig. 4:**
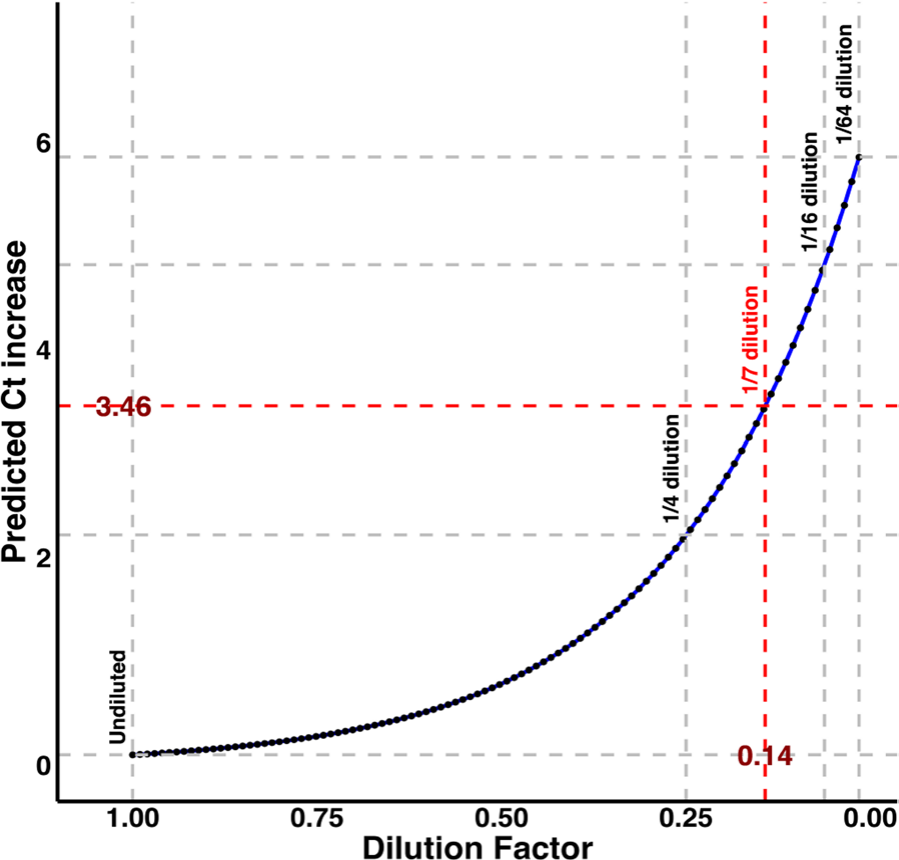
Predicted PRRSV RT-qPCR cycle threshold (Ct) value increases by dilution factor. The red broken line represents the predicted Ct increase (3.46) of the 1/7 dilution level (or pooling one PRRSV RT-qPCR-positive daily TF sample with 6 other PRRSV RT-qPCR negative TF samples)

The least-squares mean increases in PRRSV RT-qPCR Ct for the 1/4, 1/16, and 1/64 dilutions were 2.37 (1.50, 4.00), 4.52 (3.55, 6.00), and 6.10 (5.06, 7.00), respectively. Observed and fitted values were comparable (Additional file 1: Figure S1). The fitted model predicted a 3.46 Ct increase at the 1/7 (0.14) dilution (Fig. 4), meaning a PRRSV-positive TF sample would exhibit a 3.46 rise in Ct when pooled with six PRRSV-negative TF samples.

**Fig. 5:**
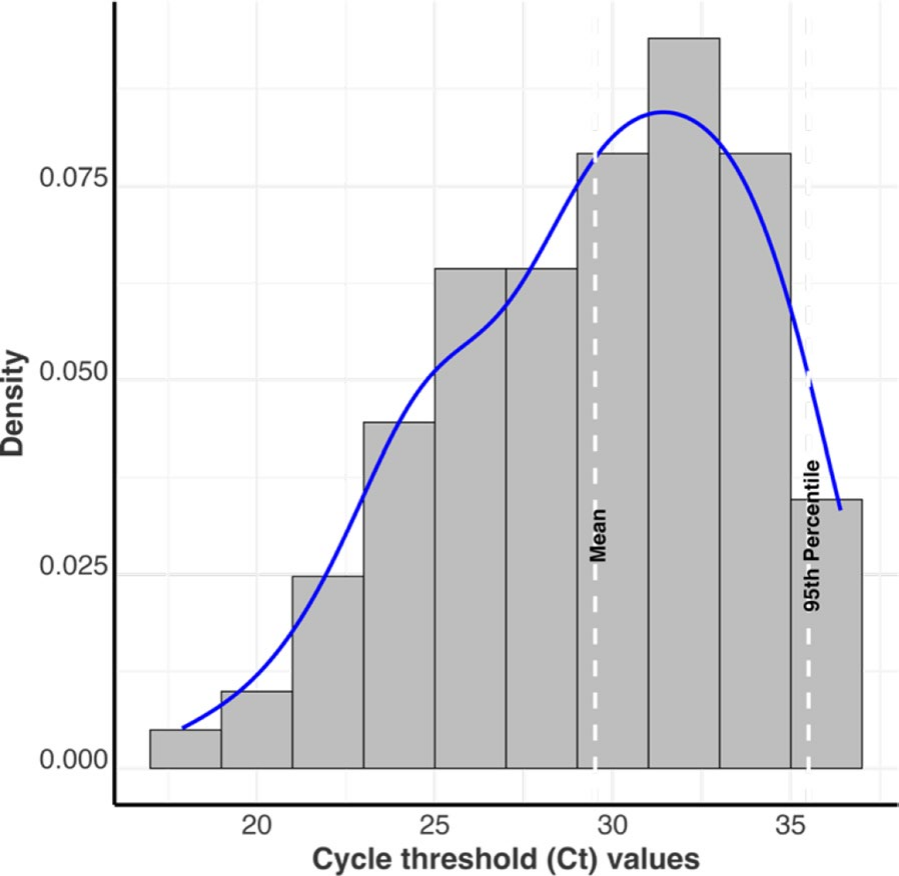
The distribution of PRRSV RT-qPCR cycle threshold (Ct) values of postmortem tongue fluids samples submitted to the Iowa State University veterinary diagnostic laboratory from growing pig herds in the US from 2022 to 2024. The blue line is the probability density curve of the distribution of Cts

The distribution of the Ct values of non-pooled TF submitted to the Iowa State University veterinary diagnostic laboratory from growing pig herds in the US is shown in Fig. 5. The mean Ct was 29.5 and the 95th percentile Ct was 35.5.

Applying the prediction model (Ct increases with increasing dilution) to the empirical distribution of starting Ct values (ISU VDL PRRSV Ct for TF samples), the dilution factor below which 95% of the starting Ct values stayed below 37 is 0.35. At 1/7 dilution, 79% of the starting Cts stayed below 37 (Additional file 1: Figure S2).

### Practical implications of pooling results

**Fig. 6:**
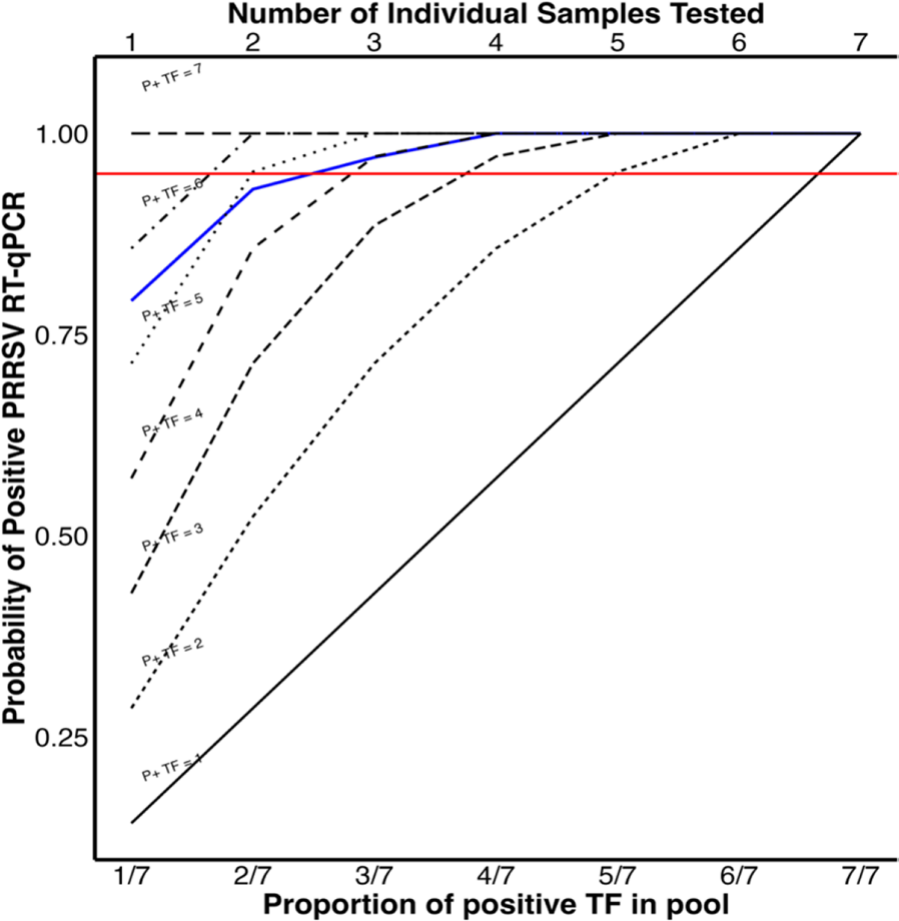
The probability of positive RT-qPCR given (1) the proportion of daily TF that are positive in a weekly pool (primary x-axis and the blue colored curve) and (2) the number of individual daily TF tested (secondary x-axis (top) and all the black colored curves). The individual black colored lines are the different scenarios where only 1 (P + TF = 1) to 7 (P + TF = 7) daily TF are positive in that week

If only one random day out of 7 herd days has PRRSV RT-qPCR positive TF, the probability of sampling the only positive day (out of a possible 7) is 14.29%. If TF samples are collected from 2, 3, 4, 5, 6 or 7 days and all individually tested, the probabilities of detection are 28.57%, 42.86%, 57.14%, 71.43%, 85.71%, 100.00%. If one weekly pool is tested instead in this same scenario, the probability of a positive test is 79% (Fig. 6). When more than 2 random days in a week are positive, the probability of a positive PRRSV RT-qPCR goes above 95% when one weekly pool is tested.

### Mortality and pathogen detection

**Fig. 7:**
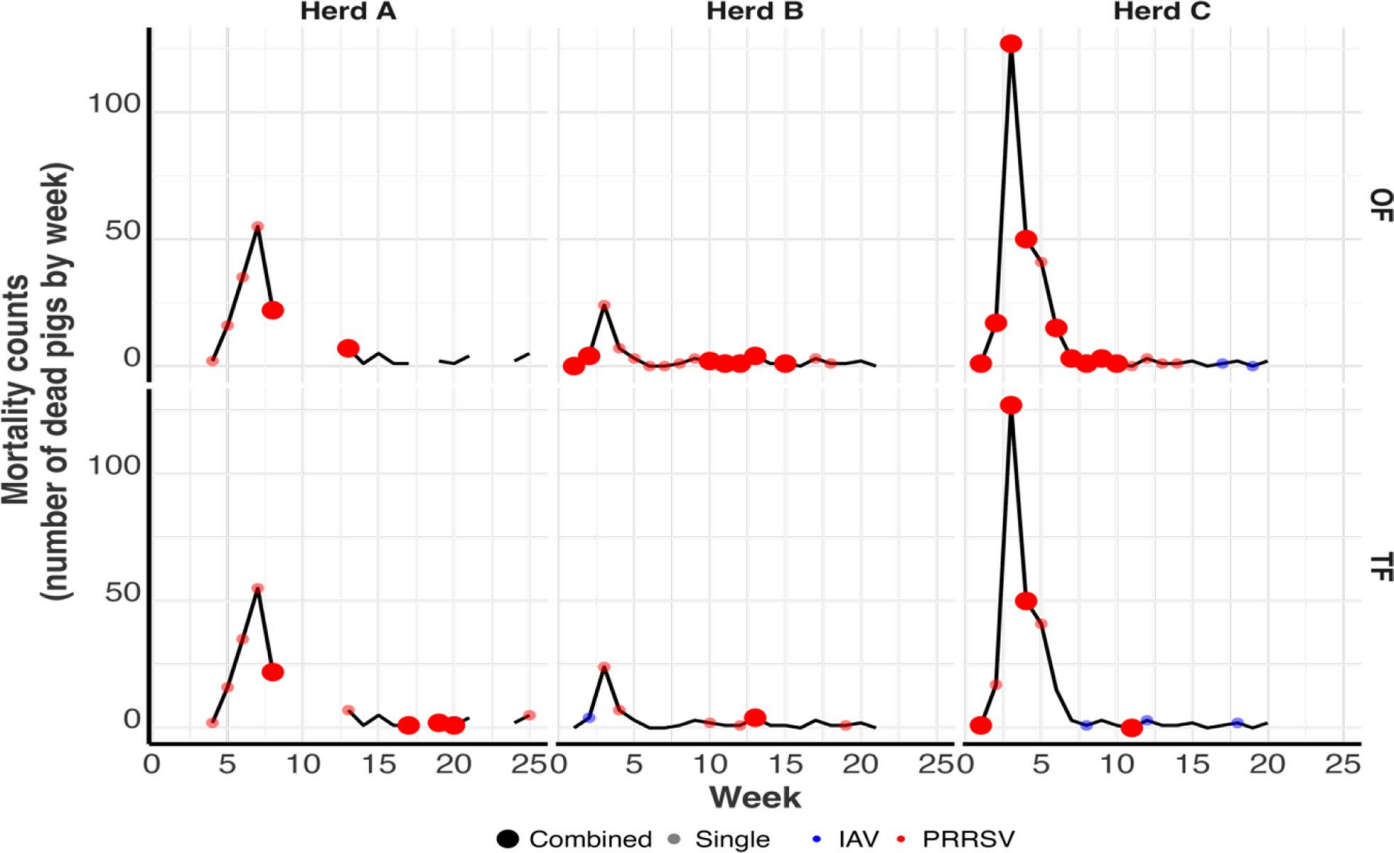
Weekly mortality across all three herds (Herds A, B, and C) showing pathogen detections in oral fluids (OF: upper panel of three plots) and postmortem tongue fluids (TF: lower panel of three plots). The smaller red circles represent weeks where only PRRSV was detected, the blue circles represent weeks where only IAV was RT-qPCR detected, and the bigger red circles represent weeks where both pathogens were detected. The lines break in weeks where no samples were submitted

For all three herds, weekly mortality rates were highest in the first few weeks post-placement, regardless of the disease pathogen status (Fig. 7). The pigs sourced.

For OF samples specifically (Table 3), the weekly mortality rate was 10.07 when only PRRSV was detected and 13.84 when PRRSV and IAV were detected. The weekly mortality rates were significantly lower when IAV alone was detected (0.25) or when none of both pathogens was detected (1.32).

**Table 3:** Weekly mortality rates across all three herds when no pathogen (Negative) was detected, when PRRSV alone was detected by RT-qPCR (PRRSV-only), when IAV alone was detected by RT-qPCR (IAV-only), and when PRRSV and IAV were detected by RT-qPCR (PRRSV and IAV) in oral fluids (OF) samples or postmortem tongue fluids (TF) samples

Sample type	Pathogens detected	Weekly mortality rate (95% confidence interval)
OF samples	PRRSV only	10.07 (1.99, 50.97)^b^
	PRRSV and IAV	13.84 (2.73, 70.00)^c^
	IAV only	0.25 (0.01, 4.90)^a^
	None	1.32 (0.25, 7.05)^a^
TF samples	PRRSV only	16.95 (4.94, 58.13)^b^
	PRRSV and IAV	16.52 (4.79, 56.96)^b^
	IAV only	1.46 (0.34, 6.31)^a^
	None	1.15 (0.30, 4.43)^a^

Groups with different letters are statistically different (*p* < 0.05)

For TF samples specifically (Table 3), the weekly mortality rates were 16.95 when only PRRSV was detected and 16.52 when PRRSV and IAV were detected. The weekly mortality rates were significantly lower when IAV alone was detected (1.46) or when none of both pathogens was detected (1.15). Together, weekly mortality rates were significantly higher when PRRSV RNA was detected by RT-qPCR either alone or alongside IAV.

## Discussion

The overarching goal of this study was to assess the suitability of TF as a sample type for swine pathogen surveillance in U.S. growing pig populations, building on its proven utility and efficiency in breeding herds [5, 6]. To evaluate the performance of TF samples, specific surveillance outcomes for a single pathogen (PRRSV) were compared to oral fluids, the industry-accepted standard for surveillance in growing pig herds. On average, as can be seen from the PRRSV RT-qPCR Ct values, the viral loads were significantly higher in TF samples compared to OF samples even though the OF samples were tested individually and TF samples in weekly pools. This finding aligns with previous reports of relatively high PRRSV concentration in TF compared to other sample types obtained from the same population of animals [15].

The relatively high viral load of PRRSV in TF compared to OF was not observed with IAV. This difference may be due to the likely presence of PRRSV in serum that flows out from a severed tongue, along with other fluids in the mouth. In contrast, IAV primarily resides in the respiratory system of pigs, and consequently, similar IAV amounts in both OF and TF samples are expected.

In weeks where both TF and OF were submitted, the Bayesian latent class model estimated a higher sensitivity but lower specificity in OF samples compared to TF for IAV and PRRSV. Taken together, the TF and OF sample types had moderate diagnostic accuracy values for both pathogens, agreeing with a previous report of comparable PRRSV detection in TF and OF samples from weaners [16]. The similar detection rates for PRRSV and IAV in both sample types, despite conducting only one RT-qPCR test per week for TF, further highlight the cost-effectiveness and reasonably high herd sensitivity of TF samples—two critical features for maintaining sustainable, best-practice surveillance. Previous studies have underscored the importance of frequent testing to the success of monitoring and surveillance programs in swine populations [17–20]. To enable regular sampling, an ideal sample type or testing strategy (e.g., pooling or not) should be capable of detecting the pathogen early and at minimal cost. This is particularly relevant for U.S. growing pig populations, where biosecurity investments are typically lower, and disease outbreaks are more common compared to breeding herds [21–23]. The findings from this study support that TF samples are well-suited for robust surveillance programs targeting PRRSV and IAV.

The availability of TF samples depends on the presence of mortalities. In this study, some weeks had no mortalities, underscoring the need for swine pathogen surveillance in US growing pig populations to incorporate one or more antemortem sample types alongside postmortem tissues for effective herd health monitoring. OF remains a viable alternative to TF for PRRSV and IAV surveillance. In growing pig herds, clinical signs or productivity issues often prompt surveillance, with mortality being a common trigger. In this context, TF is ideal, as deceased pigs are a risk-based population likely to harbor the outbreak pathogen or parts of it. Dead animals are also expected to have a higher pathogen prevalence and pathogen level per pig compared to the general population of living animals. In the early stages of an outbreak where prevalence is low and most pigs are non-clinical, febrile pigs are more likely to lie down at the back of the pens and not willing or able to interact with sampling ropes; surveillance programs in such situations will need to be supplemented with other sample types, such as TF.

Associations were investigated between mortality and swine pathogen detection in TF compared to the same association in OF samples. The longitudinal detection of multiple swine pathogens in OF from growing pigs and their association with productivity parameters have been documented previously [24, 25]. In this study, the association between mortality and weekly RT-qPCR detection of PRRSV and IAV were comparable in TF and OF sample types with significantly higher mortality rates occurring in weeks where PRRSV was detected by RT-qPCR. Cohen's Kappa analysis showed fair agreement in the RT-qPCR detection rates for PRRSV and IAV across both sample types. The discrepancy in inter-rater agreement is further emphasized by the diagnostic performance of TF samples, using RT-qPCR outcomes from OF as the reference. It is plausible that cross-sectionally obtained OF samples might fail to capture sick or positive pigs. This limitation arises from the fact that OF sampling relies on animals’ willingness to interact with the sampling rope, meaning the absence of positive OF samples in any given week does not conclusively indicate the absence of pathogens. Likewise, several factors may have affected TF testing outcomes, including an insufficient number of tissues sampled or sampling tongues from fewer animals than the actual number of mortalities.

The successful sequencing of the PRRSV ORF-5 gene from both TF and OF provides valuable insights into the genetic diversity of circulating viral strains. Understanding the genetic landscape of PRRSV within a herd can guide practitioners in making informed decisions regarding herd vaccination protocols and management practices [26–29]; ultimately leading to better health and productivity outcomes. The inverse relationship between Ct values and the probability of obtaining PRRSV sequences is not new [30, 31].

Even though, the observed rates of successful sequencing were higher in TF compared to OF, there was no statistically significant difference in the success rates of sequencing when the Ct of the samples were controlled for. Nonetheless, as we have seen in this study and in a previously published article [15], the lower Ct observed in TF is inherent with the sample type. Thus, the better performance of TF samples relative to OF samples regarding sequencing is actual and not coincidental. Notably, a substantial number of the tongue tissues submitted were diluted with PBS to increase sample volume, which may have underestimated the perceived advantage of TF over OF samples. Additionally, there was a time-lapse and one or more freeze–thaw cycles between initial RT-qPCR testing and sequencing, potentially resulting in higher Cts at the time of sequencing than currently reported.

In this study, although postmortem tongue tissues were collected daily, they were tested in pools. It is therefore important to measure how this testing strategy may have affected the RT-qPCR outcomes of the study or how much sensitivity may have been lost from not testing the daily samples individually. Given that resources are limited, and every dollar counts in swine enterprises that rely on efficient resource use to stay viable, this approach raises important considerations. For pathogens like PRRSV, which can persist at low prevalence over extended periods, testing representative units of any sample type as frequently as necessary is cost-prohibitive and unrealistic, prompting the question of whether weekly pooling of an already composite sample type is effective.

With pooling, there are significant benefits of achieving broader herd coverage, even though this may dilute the concentration of the target analyte. By showing that pooled TF samples can maintain PRRSV RT-qPCR detection sensitivity while conserving resources, this study suggests a sustainable and cost-effective approach for swine practitioners to monitor pathogens in growing herds. For TF samples, it is more cost-effective to test multiple daily (if mortalities are available) aggregated TF as one weekly pool than pick and individually-test fewer specific days to represent the entire week. As previously mentioned, using PBS to extend the volumes of daily tongue samples might have underestimated the advantages of pooling. Aggregating all the tongue tissues by week ab initio and testing this directly without several milliliters of PBS added to daily samples could have improved the outcome of the pooling study.

There were instances where not all daily mortalities were sampled for tissues, or none were sampled at all; this may have affected the overall performance assessment of the TF sample type. Addressing these procedural aspects would strengthen the diagnostic performance of TF samples in real-world settings and enhance their value in pathogen surveillance. The latent class model relied on the assumption that the two sample types are conditionally independent, given the latent infection status. If there is residual dependence between OF and TF test results beyond what is accounted for by the latent classes, this could bias the estimates of sensitivity and specificity.

Overall, the findings suggest that with optimized pooling and sampling methods, TF samples provide a viable and sustainable option for swine health monitoring. Building on the findings of this study, future research should broaden the scope of pathogens and diagnostic approaches investigated in TF. For instance, evaluating the diagnostic performance of TF for additional viral and bacterial pathogens would help to clarify its utility in a comprehensive swine health surveillance program. Moreover, advanced techniques such as next‐generation sequencing (NGS) and metagenomic analyses applied to TF could reveal a wider spectrum of circulating pathogens, including emerging or unexpected pathogens. These expanded applications would not only strengthen our understanding of swine disease ecology but also enhance early detection capabilities and inform more effective control strategies.

## Conclusion

Tongue fluids (TF) sampling can complement or substitute oral fluids (OF) sampling in surveillance of PRRSV and IAV. For PRRSV surveillance, significantly lower Cts or higher viral titers were observed in TF samples compared to OF samples, thus making the sample a more suitable option for PRRSV sequencing. Notably, OF samples did not surpass TF samples in most of the metrics assessed, despite being tested in smaller units compared to the aggregated weekly pools used for TF samples. Testing aggregated TF samples as weekly pools is cost-effective.

Testing aggregated TF samples on a weekly basis proves to be a cost-effective approach, offering swine practitioners a practical and sustainable method for pathogen surveillance. While TF can be collected from one or more tongue tissues, practitioners are encouraged to combine as many tongue tissues from daily mortalities as possible to maximize the diagnostic value of this sample type. Aggregating more tissues ensures that the resulting fluid sample sent for laboratory testing allows for a more representative assessment of the herd’s health status. Future investigations into broader pathogen panels and metagenomic applications in TF samples will further unlock its potential as a key diagnostic and surveillance tool in swine production.

## Methods

### Overview

Three herds of growing pigs from two production systems were monitored weekly for PRRSV and IAV from nursery placement until market. Two herds (A and B) were sourced from PRRSV-positive stable sow farms. The third herd (C) was sourced from a PRRSV-positive unstable farm that had just experienced a PRRSV outbreak. Herds B and C were given PRRSV modified live virus (MLV) vaccine at weaning. From each of the three herds, OF (n = 6) was collected weekly from 6 pens following the American Association of Swine Veterinarians’ recommendation for PRRSV surveillance in growing pig herds (Holtkamp et al., 2021), tested individually for PRRSV, and tested in 2 pools of 3 OF for IAV. Each pig pen across all three herds contained between 30 to 60 pigs. TF was collected daily and tested as one weekly pool for PRRSV and IAV. All samples were tested by RT-qPCR at the Iowa State University Veterinary Diagnostic Laboratory (ISU VDL). Twenty-two sets of individual (non-pooled) RT-qPCR positive TF and OF across all three herds, paired by week, were sent for Sanger sequencing of the PRRSV ORF-5 region. PRRSV-positive TF samples were fourfold serially diluted with PRRSV negative TF samples in the ratios “Undiluted”, 1/4, 1/16, and 1/64, to assess the effect of pooling on PRRSV RNA detection by RT-qPCR. Associations between weekly mortalities and the RT-qPCR detection of the monitored swine pathogens were also evaluated.

### Study herd and samples

#### Eligibility

Only herds willing to share laboratory and productivity data for the entire growing phase and able to give the study team access to the farm premises for sampling whenever required were enrolled in the study.

#### Study population

Three herds of pigs (A, B, and C) sourced from PRRSV-positive sow herds belonging to two production systems (1 and 2) in the Midwestern USA were enrolled in the study. The IAV statuses of the study herds were not considered in the recruitment of the herds. Herd A belonged to production system 1, while herds B and C belonged to production system 2. On the weeks of weaning or placement, sow herds from which Herds A and B were sourced were PRRSV-positive stable (AASV status 2vx). In contrast, Herd C was sourced from a PRRSV-positive unstable high-prevalence sow herd (AASV status 1A). Production system 2 vaccinates piglets with the PRRSV-modified live virus vaccine at weaning (21 days of age). Herd A had about 600 pigs total at nursery placement, while Herds B had about 1100 pigs and Herd C about 800 pigs.

Every week in all three study herds, daily aggregated tongue tissues and 6 OF samples (cross-sectionally collected on one day during the week from 6 pens geospatially) were collected and sent to the ISU VDL for RT-qPCR testing.

#### Sample size

The unit of analysis is the herd week. The study aimed to have at least 40 of the planned 75 weeks (25 wean-to-market weeks across all three herds) having at least one positive RT-qPCR result. Forty paired weekly samples provide at least an 80% power to detect a difference of 25% in positivity between the TF and OF samples (assuming the probability of PRRSV RNA RT-qPCR detection in truly positive OF and TF samples are 85% and 60%, respectively).

#### Sample collection

**Fig. 8:**
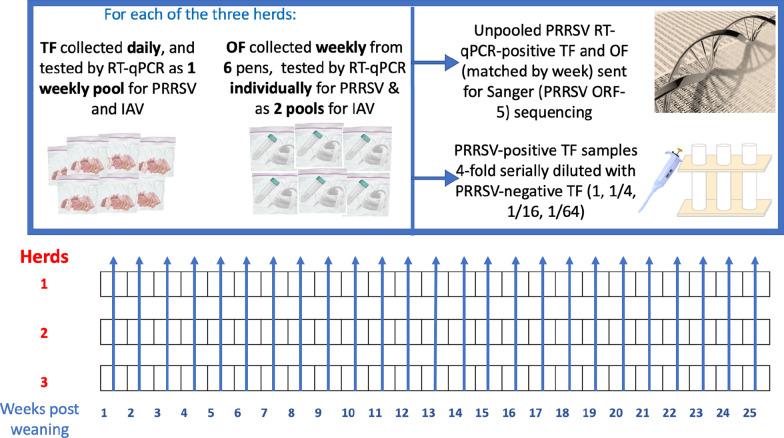
Study schematic

Tongue tip tissues (approximately 1 inch each) from the daily mortalities were collected with scissors and forceps, placed in a Ziploc bag, and stored under −20 °C until sample submission. Tongue fluids were extracted from each bag of tissues and tested by RT-qPCR. In any instance where there was an insufficient amount (≤ 1 mL) of fluids from the thawed tongue tissues, 1 mL of PBS was added to the bag of tissues, and the contents were then wrung into a 5 mL conical tube (Corning Science Mexico S.A. de C.V., Tamaulipas, Mexico) (Fig. 8). Oral fluids were obtained from 6 geographically spaced pens. An untreated cotton rope was tied to the side railing of each pen to be sampled, low enough to be accessed by the smallest pig in the pen. The pigs were allowed to voluntarily interact with the sampling rope for about 30 min, after which oral fluids were wrung from the ropes into a 5 mL conical tube using a Ziploc bag. Records of mortalities were also obtained for all three pig herds.

### Laboratory tests

#### RT-qPCR

For every study week, all daily TF collected for that study week were RT-qPCR tested as one pool for PRRSV and IAV. OF samples were collected individually per pen from a total of 6 pens and tested individually by RT-qPCR for PRRSV, and in 2 pools of 3 OF for IAV. All RT-qPCR tests were done at the ISU VDL. RT-qPCR positivity and cycle threshold (Ct) values were obtained from these tests. Only samples with Ct values less than 37 were reported by the VDL as positive. For TF pools that were RT-qPCR positive for PRRSV, the component samples making up those pools were submitted to the VDL for RT-qPCR testing. The end goal was to obtain individual candidate samples for onward Sanger sequencing and to assess the dilution effect on PRRSV RT-qPCR detection.

#### Sequencing

The Sanger sequencing of the PRRSV ORF-5 region in TF and OF samples matched by week was evaluated. The outcomes of interest were sequencing success (yes or no), and the changes in the PRRSV variant present over time and across the sample types. This was also done at the ISU VDL.

#### Pooling

Three individual PRRSV-positive TF samples were fourfold serially diluted with PRRSV-negative TF samples to obtain undiluted, 1/4, 1/16, and 1/64 dilution levels.

**Fig. 9:**
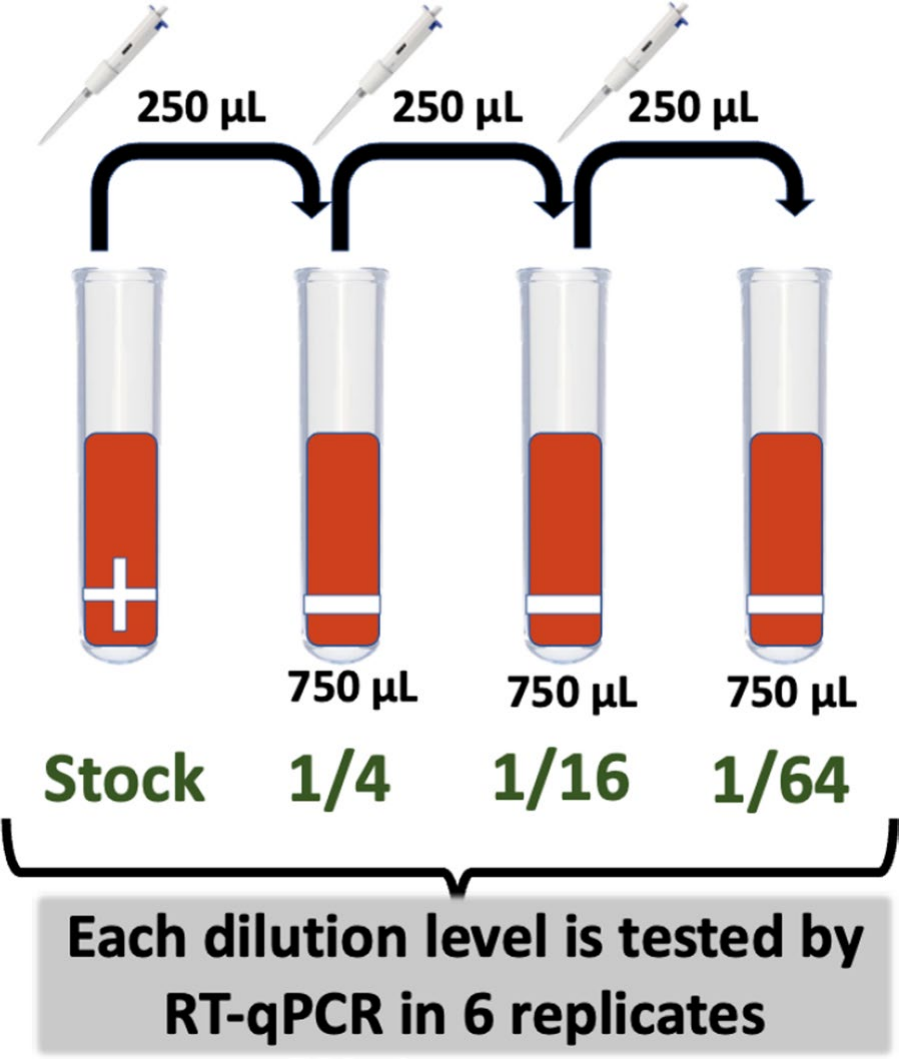
Four-fold serial dilution of PRRSV RT-qPCR-positive tongue fluid samples with negative samples

PRRSV-negative TF samples were obtained, pooled into a 50 mL conical tube, vortexed, and tested by RT-qPCR to confirm that they were PRRSV RNA negative. 750 μL of the pooled negative samples were aliquoted into 9 5 mL conical tubes (3 5 mL tubes for diluting each positive TF sample). Each positive TF sample was vortexed at 3200 revolutions per minute for approximately 10 s, and 250 μL transferred to one tube (A) already containing 750 μL of negative TF; tube A also vortexed for about 10 s, and 250 μL transferred to another tube (B) containing 750 μL of negative TF. Tube B was also vortexed similarly, and 250 μL of the resulting mixture was transferred to a third tube C containing 250 μL (Fig. 9). All samples and dilution levels were submitted to the earlier mentioned VDL for PRRSV RT-qPCR, with each being tested in 6 replicates.

### Data analysis

#### RT-qPCR detection and Ct values

Descriptive plots and tables were used to demonstrate differences in the RT-qPCR detection rates and Ct values between weekly pooled TF and weekly averages of OF for PRRSV and IAV. The differences in detection rates were only assessed for weeks when both sample types were submitted for laboratory testing. Mixed-effects regression models were used to evaluate differences in Ct values, with the response variable being the Ct value, the sample type and pathogen used as fixed effects, and the herd was treated as a random effect. Post-hoc comparisons were performed using Tukey's test with Sidak-adjusted p-values, assuming 5% type 1 error (significance level).

#### Diagnostic performance of both sample types

A herd week was considered positive for a swine pathogen and sample type if at least one sample tested RT-qPCR positive for that pathogen using RT-qPCR. For all weeks where both TF and OF submissions were available, two-by-two contingency tables were constructed to summarize the RT-qPCR detections of IAV and PRRSV. Since neither TF nor OF serves as a gold standard for detecting either of the assessed pathogens, a latent‐class approach was utilized [32]. Each of the 43 farm weeks wherein both sample types were submitted was assumed to have a latent (unobserved) infection status for IAV and another for PRRSV. The probability of infection (and by extension, prevalence) was modeled to vary by farm through random intercepts on the logit scale. The prevalence for IAV and PRRSV on farm $f$ was modeled as [31]:

$$logit\left( {p_{IAV} ,f} \right) = \mu_{IAV} + b_{IAv} ,f, b_{IAV} ,f \sim N\left( {0,\tau_{IAV}^{2} } \right);$$

$$logit\left( {p_{PRRSV} ,f} \right) = \mu_{PRRSV} + b_{PRRSV} ,f, b_{PRRSV} ,f \sim N\left( {0,\tau_{PRRSV}^{2} } \right);$$

Here, ${\mu }_{IAV}$ and ${\mu }_{PRRSV}$ represent the global mean logit prevalence for IAV and PRRSV, respectively, while ${\tau }_{IAV}^{2}$ and ${\tau }_{PRRSV}^{2}$ denote the variances of farm-level prevalence.

For each farm‐week $i$, latent infection indicators were drawn from a Bernoulli distribution with probabilities ${p}_{IAV},{f}_{\left(i\right)}$ and ${p}_{PRRSV},{f}_{\left(i\right)}$. The test outcomes for each sample type and pathogen were modeled as Bernoulli variables conditional on the latent state, with unknown sensitivity and specificity.

Weakly informative Beta (2, 2) priors for diagnostic performance parameters (Sensitivity and Specificity) were used to reflect moderate prior uncertainty without imposing strong assumptions. Normal priors with mean 0 and precision 0.5 were used for the logit of farm‐level prevalence, and a uniform (0,5) prior was used for the farm‐level standard deviation.

The model was implemented in R using the rjags package [33]. Four parallel Markov chain Monte Carlo (MCMC) chains were run with 2000 iterations of burn‐in, followed by 10,000 sampling iterations each (thinning by 2). Convergence was assessed using trace plots and the Gelman–Rubin statistic ($\widehat{R}$) [34]. Posterior summaries (mean, median, 95% credible intervals) were extracted for each parameter.

#### Sanger sequencing outcomes

Sanger sequencing success rates for the PRRSV ORF-5 gene were compared between both sample types. Mixed effects regression models were also used to characterize statistical differences in Sanger sequencing success between both sample types. The binary variable (successful or not) was the outcome variable; the sample type and Ct values were used as fixed effects, and the herd was used as a random effect.

#### PRRSV RT-qPCR Ct changes with dilution

The relationship between dilution levels and Ct increases was modeled as a linear effect of the logarithm of the dilution factor. This approach assumes that changes in dilution levels correspond to predictable increases in Ct values. A Gaussian distribution with a log link function was applied to capture this relationship, and the model was implemented using the *glm()* function in base R.

The predictive model built for the effect of dilution on Ct differences is given by:

$$\Delta Ct=\beta 0 +\beta 1* log(Dilution Factor)+\epsilon$$

where: $\Delta Ct$ is the predicted increase in Ct value due to dilution, $\beta 0$ is the intercept, representing the baseline shift in Ct, $\beta 1$​ is the coefficient for the log of the dilution factor, and $\epsilon$ is the normally distributed error term.

#### Implication of pooling TF on PRRSV RT-qPCR detection

This was done in two steps:

##### Obtaining an empirical distribution of baseline TF Ct values

An empirical distribution of TF PRRSV RT-qPCR Ct values was constructed using retrospective data from the Iowa State University Veterinary Diagnostic Laboratory (ISU VDL). Data included non-pooled TF samples from growing pig herds submitted between 2022 and 2024 (n = 102). Using the *sample()* function in base R, 10,000 Cts were randomly sampled (with replacement) from this dataset to create an empirical distribution representative of TF Ct values in U.S. growing pig populations.

##### Applying the predictive model to determine detection thresholds

The earlier mentioned predictive model of Ct changes with dilution was applied to the empirical distribution of starting Ct values to predict total Ct values at various dilution levels. By repeatedly sampling from the empirical distribution and applying the predictive model, the minimum dilution factor was identified at which 95% of combined Ct values exceeded the threshold of 37, indicating a failure to detect PRRSV RNA at that dilution level.

Finally, the probability of detecting PRRSV RNA in weekly pooled TF samples was compared to that of daily testing of individual TF samples to assess the practical implications of pooling TF for PRRSV surveillance.

#### Pathogen detection and mortality

The association between weekly pathogen detection and mortality was characterized using mixed-effects Poisson regression models, with separate models for TF and OF. Weekly mortality counts served as the response variable, while pathogen detection (PRRSV and/or IAV) was included as a fixed effect. The log of the weekly pig inventory was included as an offset. The herd was modeled as a random effect to account for herd-specific sources of variation. All statistical analyses and visualizations were conducted using the lme4 [35] and the ggplot2 [36] packages, respectively, on the R statistical software [37].

## Supplementary Information

Additional file 1

Additional file 2

## Data Availability

Any data not here presented will be made available upon reasonable request.
