# Supplementary material for: Evaluating postmortem tongue fluids as a tool for monitoring PRRSV and IAV in the post-wean phases of swine production
Source: Porcine Health Manag. 2025 Apr 7;11:18. doi: 10.1186/s40813-025-00432-x (PMC11978129; doi:10.1186/s40813-025-00432-x)
Supplement: Supplementary file 1 — Additional file 1 [file 40813_2025_432_MOESM1_ESM.docx]

**
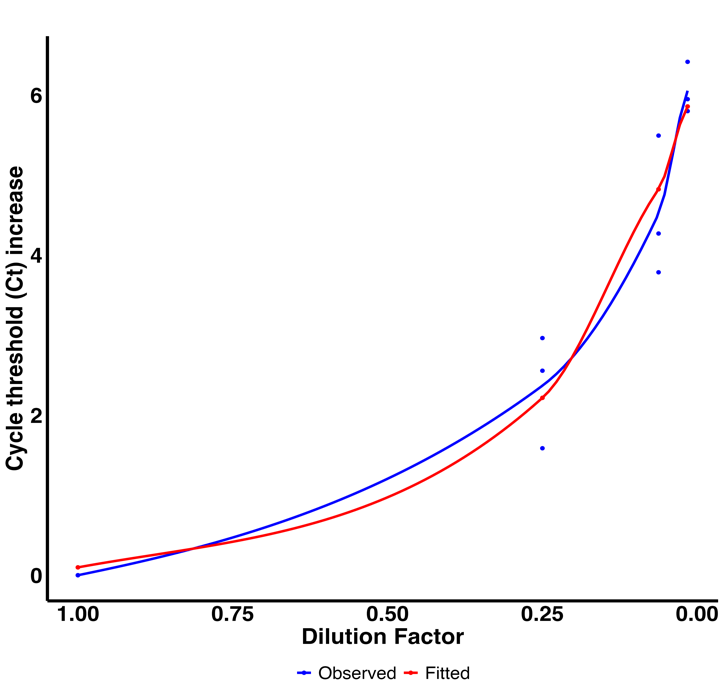
SUPPLEMENTARY FIGURES**

**Figure S1. The** PRRSV RT-qPCR cycle threshold (Ct) increases **by dilution factor. The blue dots and line are the observed Ct values from the experiment while the red dots and line are the fitted values from the statistical model.**

**
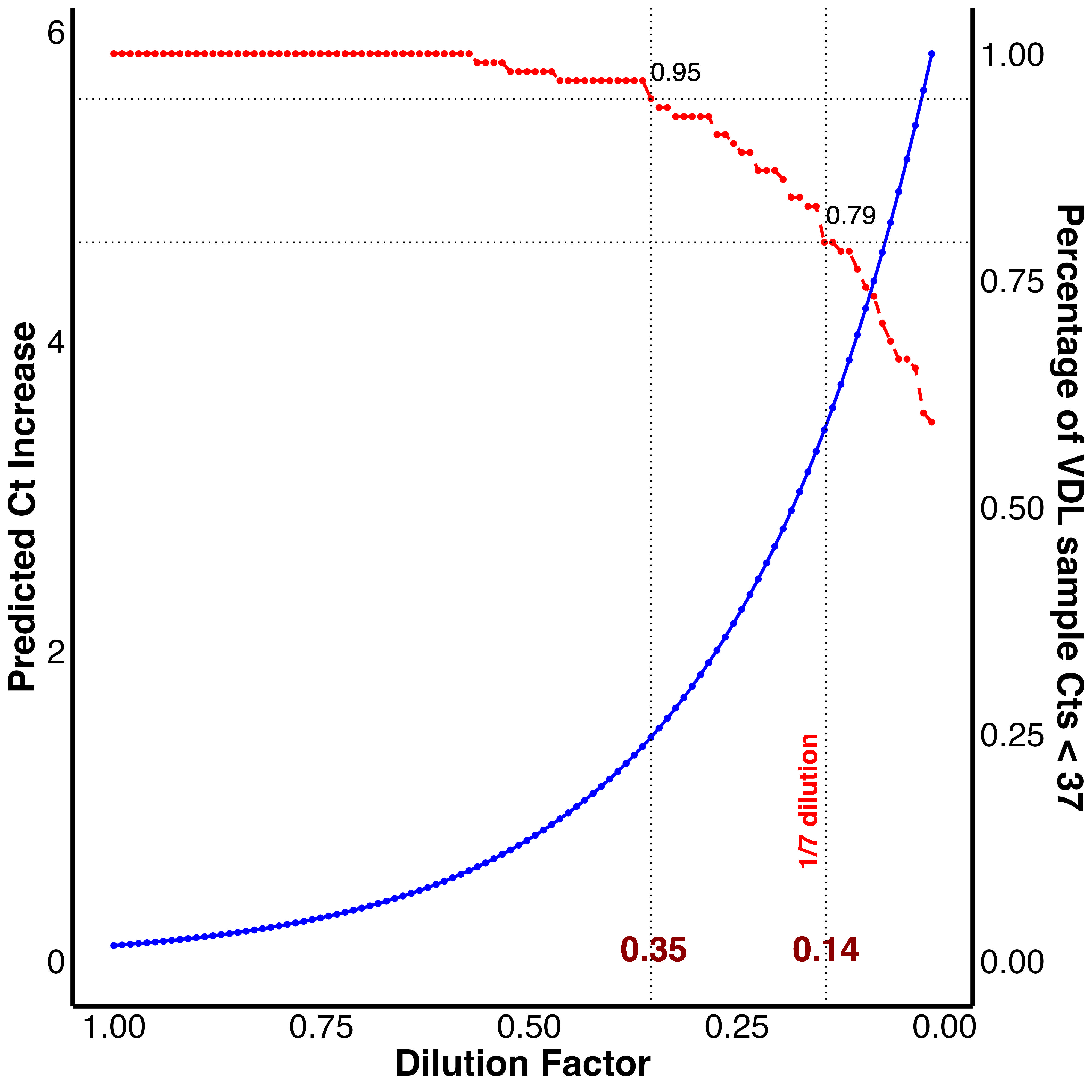
**

**Figure S2**. PRRSV RT-qPCR cycle threshold (Ct) increases (primary y-axis and the blue curve) and the percentage of starting Ct values (ISU VDL PRRSV Ct for TF samples) that stay positive (secondary y-axis and the red curve) with increasing dilution (x-axis). The dotted lines represent the dilution factor at which 95% of starting Cts stay positive (0.35) and the percentage of starting Cts that stay positive at 1/7 or 0.14 dilution (79%).
