# Supplementary material for: Evaluating postmortem tongue fluids as a tool for monitoring PRRSV and IAV in the post-wean phases of swine production
Source: Porcine Health Manag. 2025 Apr 7;11:18. doi: 10.1186/s40813-025-00432-x (PMC11978129; doi:10.1186/s40813-025-00432-x)
Supplement: Supplementary file 2 — Additional file 2 [file 40813_2025_432_MOESM2_ESM.docx]

**Table S1**. PRRSV ORF-5 Sanger sequencing results and reverse transcription quantitative polymerase chain reaction (RT-qPCR) cycle threshold (Ct) values from weekly matched oral fluids (OF) and postmortem tongue fluids (TF) collected from three growing pig groups (Herds A, B, and C). ORF-5 RFLP patterns and viral lineage classification are also included for successfully sequenced samples.

| **Farm** | **Sample**  **type** | **Study**  **week** | **RT-qPCR Ct** | **Sequencing**  **Success**  **(1 = yes, 0 = no)** | **ORF-5 and**  **lineage** | **Type** |
| --- | --- | --- | --- | --- | --- | --- |
| Herd A | OF | 4 | 27.1 | 1 | 1-4-4 L1A | Wild-type |
| Herd A | TF | 4 | 19.4 | 1 | 1-4-4 L1A | Wild-type |
| Herd A | OF | 5 | 27.2 | 1 | 1-4-4 L1A | Wild-type |
| Herd A | OF | 5 | 27.9 | 1 | 1-4-4 L1A | Wild-type |
| Herd A | TF | 5 | 22.1 | 1 | 1-4-4 L1A | Wild-type |
| Herd A | TF | 5 | 21.5 | 1 | 1-4-4 L1A | Wild-type |
| Herd A | OF | 6 | 27 | 1 | 1-2-4 L1A | Wild-type |
| Herd A | OF | 6 | 26.1 | 1 | 1-4-4 L1A | Wild-type |
| Herd A | OF | 6 | 28.7 | 1 | 1-4-4 L1A | Wild-type |
| Herd A | TF | 6 | 17.9 | 0 | - | - |
| Herd A | TF | 6 | 23.1 | 1 | 1-4-4 L1A | Wild-type |
| Herd A | TF | 6 | 23.5 | 1 | 1-4-4 L1A | Wild-type |
| Herd A | OF | 7 | 31.5 | 0 | - | - |
| Herd A | TF | 7 | 20.7 | 1 | 1-4-4 L1A | Wild-type |
| Herd A | OF | 8 | 33.7 | 0 | - | - |
| Herd A | OF | 8 | 32.4 | 0 | - | - |
| Herd A | OF | 8 | 31.6 | 1 | 1-4-4 L1A | Wild-type |
| Herd A | TF | 8 | 25.2 | 1 | 1-4-4 L1A | Wild-type |
| Herd A | TF | 8 | 29.1 | 0 | - | - |
| Herd A | TF | 8 | 31.5 | 0 | - | - |
| Herd B | OF | 3 | 31.5 | 1 | 1-1-2 L8C | Vaccine-like |
| Herd B | TF | 3 | 34.2 | 1 | 1-1-2 L8C | Vaccine-like |
| Herd B | OF | 4 | 34.2 | 0 | - | - |
| Herd B | OF | 4 | 34.8 | 0 | - | - |
| Herd B | TF | 4 | 34.8 | 0 | - | - |
| Herd B | TF | 4 | 34.6 | 1 | 1-3-2 L8C | Vaccine-like |
| Herd B | OF | 10 | 27.6 | 1 | 1-3-2 L8C | Vaccine-like |
| Herd B | TF | 10 | 31.1 | 1 | 1-3-2 L8C | Vaccine-like |
| Herd B | OF | 12 | 35.9 | 0 | - | - |
| Herd B | TF | 12 | 24.8 | 1 | 1-1-2 L8C | Vaccine-like |
| Herd B | OF | 13 | 36.3 | 0 | - |  |
| Herd B | TF | 13 | 32.8 | 0 | - |  |
| Herd C | OF | 1 | 32 | 0 | - |  |
| Herd C | OF | 1 | 30.8 | 0 | - |  |
| Herd C | OF | 1 | 29.6 | 1 | 1-7-2 L1A | Wild-type |
| Herd C | TF | 1 | 24.3 | 1 | 1-7-2 L1A | Wild-type |
| Herd C | TF | 1 | 23.7 | 1 | 1-7-2 L1A | Wild-type |
| Herd C | TF | 1 | 25.2 | 0 | - | - |
| Herd C | OF | 2 | 32.4 | 0 | - | - |
| Herd C | TF | 2 | 26.8 | 1 | 1-3-2 L8C | Vaccine-like |
| Herd C | OF | 3 | 32.6 | 0 | - | - |
| Herd C | OF | 3 | 32.4 | 0 | - | - |
| Herd C | TF | 3 | 32.5 | 0 | - | - |
| Herd C | TF | 3 | 28.3 | 0 | - | - |
